# Psychosocial factors and mental work load: a reality perceived by nurses in intensive care units^1^

**DOI:** 10.1590/0104-1169.0044.2557

**Published:** 2015

**Authors:** Paula Ceballos-Vásquez, Gladys Rolo-González, Estefanía Hérnandez-Fernaud, Dolores Díaz-Cabrera, Tatiana Paravic-Klijn, Mónica Burgos-Moreno

**Affiliations:** 2 Doctoral student, Departamento de Enfermería, Universidad de Concepción, Concepción, Chile. Assistant Professor, Departamento de Enfermería, Universidad Católica del Maule, Talca, Chile; 3 PhD, Full Professor, Departamento de Psicología Cognitiva, Social y Organizacional, Universidad de la Laguna, Tenerife, Spain; 4 PhD, Full Professor, Departamento de Enfermería, Universidad de Concepción, Concepción, Chile; 5 PhD, Associate Professor, Departamento de Enfermería, Universidad de Concepción, Concepción, Chile

**Keywords:** Occupational Risks, Nursing, Occupational Health, Workload

## Abstract

**OBJECTIVE:**

To analyse the perception of psychosocial factors and mental workload of nurses who work in intensive care units. It is hypothesised that nurses in these units could perceive psychosocial risks, manifesting in a high mental work load. The psychosocial dimension related to the position's cognitive demands is hypothesised to mostly explain mental work load.

**METHOD:**

Quantitative study, with a descriptive, cross-sectional, and comparative design. A total of 91% of the intensive care unit populations of three Chilean hospitals was surveyed, corresponding to 111 nurses. The instruments utilised included (A) a biosociodemographic history questionnaire; (b) the SUSESO-ISTAS 21 questionnaire; and (c) the Mental Work Load Subjective Scale (ESCAM, in Spanish).

**RESULTS:**

In total, 64% and 57% of participants perceived high levels of exposure to the psychosocial risks Psychosocial demands and Double shift, respectively. In addition, a medium-high level of overall mental load was observed. Positive and significant correlations between some of the SUSESO-ISTAS 21 and ESCAM dimensions were obtained. Using a regression analysis, it was determined that three dimensions of the psychosocial risk questionnaire helped to explain 38% of the overall mental load.

**CONCLUSION:**

Intensive care unit nurses felt that inadequate psychosocial factors and mental work overload existed in several of the tested dimensions.

## Introduction

Nurses who work in intensive care units (ICUs) take care of people with acute and/or chronic health problems that require permanent, specialised and highly specific nursing care. In addition, the nurses perform their roles in a context where certain characteristics coexist, such as (A) direct work with vulnerable persons; (b) high level of responsibility over their tasks and the consequences of possible errors; (c) the need to confront unpredictable events, suffering, pain and death; (d) the development of critical judgement regarding the actions produced after a medical diagnosis; (e) interacting with the families of the people they take care of; and (f) maintaining a balance between work and personal life^(^ 1 ^-^ 2 ^)^.

Other work factors exist that can be damaging for these professionals, such as the organisational characteristics of the health care environment, the constant and high mental demands, routine performance issues, an insufficient number of human resources, and nocturnal and shift work. These factors can generate emotional alterations (e.g., irritability), the presence of somatic symptomatologies (e.g., migraines, gastrointestinal problems), dissatisfaction, burnout, stress (3) or mental fatigue (4). For these reasons, working conditions ^(^ 5 ^)^-and organisational, work, technological and psychosocial factors in particular-carry great importance.

Psychosocial factors are understood to be the interaction between work, workers, the environment, satisfaction with the work performed and organisational conditions. In addition, these factors may also involve the capacity of the worker, his/her needs, culture and personal situations. These factors positively and negatively influence the health, welfare and performance of the worker^(^ 6 ^-^ 8 ^)^.

The consequences of psychosocial factors on health have been explored in various models, such as the Karasek and Theorell Job Demand-Control-Social Support model ^(^ 9 ^)^ and the Siegrist Effort-Reward Imbalance models^(^ 10 ^)^. These psychosocial factors can be grouped as (a) psychological demands; (b) balance between tasks and work, family, and social time; (c) control over work; (d) social and instrumental support of colleagues and superiors, particularly quality of leadership; (f) work rewards; and (g) job security. These theoretical models have served as the basis for the development of the Copenhagen Psychosocial Questionnaire (CoPSoQ), an instrument adapted and validated in many countries, among them Chile, for the investigation, evaluation and prevention of psychosocial risks^(^ 11 ^-^ 12 ^)^.

The objective of this research fundamentally involves the analysis of psychosocial risks that affect workers, their tasks and the organisation. In the area of Nursing, studies on stress and burnout have proliferated^ (13-14)^, leaving behind other psychosocial variables, such as mental workload^(^ 15 ^-^ 16 ^)^, which is what has powered the development of this study.

Mental load is a multidimensional construct defined as the interaction between cognitive demands of a task (e.g., memory, attention), characteristics of the person (e.g., educational level, self-efficacy) and the characteristics of the situation (e.g., temporary pressure). Among its causes, the characteristics of the task must be mentioned (e.g., memory and attention demands), along with temporary pressures and work pace, functions to be performed, the degree of autonomy, and interactions with other people^(^ 17 ^)^.

The imbalance between demands of the task and workers' skills and characteristics can cause mental work overload or underload. Work overload is understood to be situations in which the worker is faced with more demands than he or she is capable of confronting. Mental underload, however, is produced in positions with few tasks and little cognitive demand (qualitative underload) and/or simple tasks with sufficient time for execution (quantitative underload). Mental demands are one of the main sources of mental load, negatively affecting perception and resulting in damaging effects on both the heath of the workers and the achievement of the organisation's objectives^(^ 17 ^-^ 18 ^)^.

The objective of this study is to analyse the perception of psychosocial factors and mental work load of nurses who are employed in ICUs. To this end, three hypotheses are proposed: (a) Nurses perceive damaging levels of psychosocial factors in their work; (b) Nurses who are employed in the ICU will have high mental work load scores; and (c) The psychosocial dimension related to the *Cognitive demands* of the position will explain the perception of mental work load more than other measures.

## Materials and methods

This descriptive, cross-sectional and comparative study design was performed with a quantitative design approach. The study population consisted of 122 nurses from three ICUs in the central-southern region of Chile who were asked to voluntarily participate in the study. Of this total, 111 nurses responded to the questionnaire (91%). Data were not obtained from the remaining 9% (11 nurses) who were on leave or were absent from their respective institutions during the data harvesting period or who did not want to participate.

For this investigation, three instruments were utilised:

- Biosociodemographic history questionnaire: Developed for this study, where variables such as age, institution, unit and work duties; execution of other functions within the unit; type of contract; and type of shift were included.

- SUSESO-ISTAS 21^(^ 12 ^)^ questionnaire (short version): Addressing the evaluation of psychosocial factors, including 20 items grouped in five dimensions: (a) *Psychosocial demands *(sensory, emotional, quantitative, and cognitive); (b) *Active work and skill development*, including influence, autonomy, feelings about the work and development opportunities; (c) *Social support within the organisation and quality of leadership*, referring to the clarity and conflict of the role performed, leadership characteristics exercised and socio-instrumental support on the part of the nurses superiors or colleagues; (d) *Rewards*, including perceived esteem, insecurity with respect to work conditions and work performed; and (e) *Double shift*, referring to the domestic and/or family quantity of work that depends on the worker and the worry that the domestic work and/or family tasks create for them. The questionnaire delivers the worker's level of perceived risk, which is classified into low, medium and high categories. This instrument was adapted, validated and standardised for Chile by the Health Authority ^(^ 19 ^)^ and includes high, medium, and low levels of psychosocial risk scores.

- Mental Workload Subjective Scale (ESCAM, in Spanish): Published by Rolo-González, Díaz-Cabrera and Hernández-Fernaud^(^ 15 ^,^ 17 ^)^ to evaluate mental work load. The 20 items and the scale are distributed into five factors: (a) Cognitive d*emands and complexity of the task *refers to the mental effort related to the performance of the job; (b) *Characteristics of the task*, concerns interruptions or distractions; (c) *Time management* collects appraisals regarding the appropriation of workers' time to perform tasks; (d) *Work pace* evaluates the organisation and planning of the worker's time and the probability of making mistakes; and (e) *Health consequences* refers to the exhaustion that performance of the position produces. The ESCAM provides an average subjective mental work load in addition to specific scores for each dimension. The scores range between 1 and 5, where 5 is high mental load and 1 is low, and includes cut-off points for the 25th, 50th and 75th percentiles. The scale has been validated for use in the Chilean population ^(^ 20 ^)^.

The principal researcher applied the instruments in the order previously described using individual interviews (15 to 20 minutes each) between May and October 2013, after informed consent was obtained. Approval was obtained from the University of Concepción School of Medicine Bioethical Committee (Chile). In addition, approval was obtained from the Ethical-Scientific Committees of the participating health institutions. The ethical principles of E. Emmanuel were considered throughout the study^(^ 21 ^)^.

For the data analysis, the *Statistical Package of Social Sciences* (SPSS)(r), version 19.0, was utilised. Statistical data cleaning was performed to verify that no multivariate outliers were present within the Mahalanobis distance^ (22)^. Subsequently, descriptive statistics were calculated (averages, medians, standard deviations and percentages) for each of the variables. Likewise, an analysis of variance was performed using MANOVA for the SUSESO-ISTAS 21 and ESCAM dimensions, considering the following categorical work variables: hospital, work unit, performance of other functions, type of contract and type of shift. Pearson's correlation coefficient was also calculated between the psychosocial factors (SUSESO-ISTAS-21) and mental load (ESCAM) dimensions. Finally, a stepwise linear regression analysis was performed in which the principle variable was overall mental load (ESCAM) and the predictor variables were the five SUSESO-ISTAS 21 dimensions.

## Results

A total of 91% of the population of nurses working in ICUs of the three regional hospitals in Chile was surveyed, corresponding to 111 nurses (eleven nurses were absent or refused to participate, which corresponded to 9% of the sample). A total of 79.3% of the participants were women, and 20.7% were men. The average age was 34.5 years (*s.d.*= 8.7, *min *= 22, *max *= 59). Of all participants, 65.8% worked in ICUs and the remaining 34.2% in intermediate care units. A total of 73.9% of participants worked with fixed contracts (permanent positions), 13.5% were substitutes and 12.6% had indeterminate (indefinite) contracts. The average number of years worked in the institution was 7.9 (*s.d. *= 7.9, *min *= 0, *max *= 35), and the average length in the current position was 6.1 years (*s.d. *= 6.3, *min *= 0, *max *= 35). A total of 85.6% worked rotating shifts**^[1]^**. A total of 45.9%, in addition to their nursing functions, performed administrative, formative or investigative activities in the UPC.

A majority of the sample perceived a high risk level in the three psychosocial factor dimensions, *psychosocial demands*, *double shift *and *social support within the organisation and quality of leadership* (see Table 1).

**Table 1 -:** Sample frequencies and percentages as functions of the level of psychosocial risk perceived in each dimension of SUSESO-ISTAS 21. Central-Southern Region, Chile, 2013 (N=111)

Dimensiones SUSESO-ISTAS 21	Nivel bajo			Nivel medio			Nivel alto	
	n	%		n	%		n	%
Exigencias psicológicas	5	4.50		35	31.54		71	63.96
Trabajo activo y desarrollo de habilidades	32	28.83		55	49.55		24	21.62
Apoyo social en la empresa y calidad del liderazgo	9	8.11		49	44.14		53	47.75
Compensaciones	30	27.03		46	41.44		35	31.53
Doble presencia	18	16.21		29	26.13		64	57.66

A total of 64% of the participants felt that their position involved a high volume of work in relation to the time available to perform it, requiring complex decision-making and constant attention, implying a high emotional wear. Likewise, 57.7% declared that, in addition to their position, they had to attend to family-domestic work demands. Additionally, 47.7% thought that their roles were not clearly defined and that the support of superiors and colleagues was insufficient. In the *Active work and skill development* and *Rewards *dimensions, medium levels of psychosocial risk were perceived.

With respect to the mental work load, the overall mental work scores (*M*= 3.47) were above the scale median, with scores ranging from 2.5 to 4.9 (see Table 2). Likewise, in the mental load profile, scores above the median point on ESCAM were observed in four of its five factors. The factors with the highest scores were *Cognitive demand and complexity of the task* and *Characteristics of the task*. The *Time management *factor presented a score that was somewhat lower than the median point of the scale.

**Table 2 -:** Descriptive statistics of overall scores and mental load dimensions perceived by ICU nurses. South-Central Zone, Chile, 2013

	M	dt	Mdn	Mín	Máx
Carga mental global	3.47	.43	3.45	2.50	4.90
Demandas cognitivas y complejidad de la tarea	3.99	.46	4.00	2.80	5.00
Características de la tarea	3.95	.56	4.00	2.00	5.00
Organización temporal	2.75	.71	2.67	1.00	5.00
Ritmo de trabajo	3.22	.79	3.00	1.70	5.00
Consecuencias sobre la salud	3.42	.86	3.50	1.30	5.00

Comparison of profiles as a function of work variables. Among the psychosocial factor dimensions, no differences related to the hospital where the survey was conducted or performance of other functions were obtained. Statistically significant interactions were detected between the profile of psychosocial factors and the unit of work (*F*(4, 106) = 2.803,* p *< .05; η^2 ^= .02), the type of contract (*F*(8, 210) = 4.199,* p *< .01; η^2 ^= .14) and the type of shift (*F*(4, 105) = 3.756,* p *< .01; η^2 ^= .12).

Differences as a function of unit were observed in the *active work and skill development *dimension: nurses who worked in intensive care (*M *= 6.45; *s.d. *= 2.54) considered that their work offered them more professional development opportunities than those in intermediate care (*M *= 7.50; *s.d. *= 2.26).

With regard to the type of contract, differences in the *Rewards *dimension were detected. Thus, the substitute or acting nurses (*M*= 6.13, *s.d.*= 2.32) perceived lower security and stability in their employment than those with indeterminate contracts (*M *= 3.00, *s.d. *= 2.35; *t *= -3.63, p < .001) or those with fixed contracts (*M *= 4.12, *s.d. *= 2.41; *t *= -2.99, p < .01).

The type of shift produces differences in the *Social Support within the organisation and quality of leadership* (*t *= 2,78, p < .01) and *Rewards* dimensions (*t *= 3.08, p < .01). Those who worked rotating shifts (*M *= 6.84, *s.d. *= 2.28) negatively valued the support that they received from their superiors and colleagues compared to those who had day shifts (*M *= 5.07, *s.d. *= 2.34). Likewise, participants with rotating work shifts (*M *= 4.55, *s.d. *= 2.53) consider that their work receives little recognition and is less stable than those who worked day shifts (*M *= 2.47, *s.d. *= 1.55). As for the mental work load profile, no differences as functions of the previously mentioned categorical variables were obtained.

Statistically significant and positive correlations existed between the dimensions and both instruments (see Table 3). The correlation between the SUSESO-ISTAS 21 *psychosocial demands *dimension and the five ESCAM dimensions, along with the correlations between the five SUSESO-ISTAS 21 dimensions and the ESCAM *Health consequences *dimension, are worthy of emphasis.

**Table 3 -:** Correlations between the psychosocial factor dimensions and mental load

		SUSESO-ISTAS-21						ESCAM			
		1	2	3	4	5		6	7	8	9
SUSESO ISTAS-21											
1	Exigencias psicológicas										
2	Trabajo activo y habilidades	.162									
3	Apoyo social y liderazgo	.260*	.410*								
4	Compensaciones	.213*	.268*	.439*							
5	Doble presencia	.299*	.137	.204*	.190^†^						
ESCAM											
6	Demandas cognitivas y complejidad	.404*	.073	.136	.257*	.303*					
7	Características de la tarea	.208^†^	.075	.183	.350*	.266*		.410*			
8	Organización temporal	.312*	.188^†^	.193^†^	.257*	.177		.281*	.240^†^		
9	Ritmo de trabajo	.235^†^	.207^†^	.199^†^	.145	.152		.120	.032	.294*	
10	Consecuencias para la salud	.379*	.254*	.362*	.391*	.371*		.325*	.226^†^	.279*	.300*

* p = .01

† p = .05

A linear regression analysis model was used in which three of the psychosocial factors that helped to explain overall mental load were obtained. *Psychosocial demands* of the position, *Compensation* and *Double shift *jointly explain 38% of the mental load (*R* ^2 ^=. 397; *R* ^2^ corrected =. 380; *F*(3,107) = 23.49; *p < *.001). The standardised β values range from 0.342 to 0.232, with tight confidence intervals (see Table 4). The condition indices are smaller than or equal to 15. *Psychosocial demands* helped to explain 10.36% (*semipartial r *= .322) of the mental load, *Rewards* alone explained 9.73% (*semipartial r *= .312) and *Double shift* independently contributed 4.79% (*semipartial r *= .219).

**Table 4 -:** Regression model with overall mental load as the principle variable and psychosocial factors as predictor variables

Variables predictoras	*B*	*SE*	β	*t*	Intervalo de confianza de 95%		Tolerancia	FIV	Índice de condición
					Inf.	Sup.			
Exigencias psicológicas	.065	.015	.342	4.288^*^	.035	.095	.886	1.129	4.28
Compensaciones	.055	.013	.322	4.155^*^	.029	.082	.937	1.067	5.16
Doble presencia	.049	.017	.232	2.920^*^	.016	.083	.894	1.119	15.06

## Discussion

The objective of this work was to analyse psychosocial and mental load factors that impact the work of ICU nurses. In general, participants perceive that their position is associated with psychosocial factors that carry high risks to health and mental work overload.

The first group of results related to psychosocial factors indicate that the majority of these workers consider that their positions require them to make many complex decisions and to maintain a fast work pace and constant attention and that their work causes emotional wear. As such, the workers indicate a high level of psychosocial risk and difficulty reconciling work life with personnel life. They also indicate that their roles are not well defined, that they have little autonomy to decide how to organise their work and that they receive little social and instrumental support from colleagues and superiors. These results support the first work hypothesis.

This appraisal of psychosocial factors varies in some dimensions as functions of the care unit where they are employed, the type of contract, and the type of shift. ICU workers feel that they have greater autonomy and more significant tasks and professional development opportunities than those in intermediate care^(^ 2 ^,^ 23 ^)^. As for the type of contract, substitute workers compared to workers with fixed and indeterminate contracts receive fewer rewards in their positions. That is to say, they perceive less stability in their employment and less recognition on the part of their superiors and experience greater worry about the changes in the tasks assigned. Additionally, those who work in rotating shifts perceive less social and instrumental support and have lower clarity of their roles than those who work day shifts.

The second table of results refers to the mental work load perceived by ICU nurses. With regard to overall scores, ICU nurses demonstrated moderate to high mental loads. Considering the specific mental load dimensions, the mental effort required to achieve optimum performance is high. In addition, the workers feel that they have high levels of distractions and interruptions and that they have to combine several tasks at the same time, factors that lead to a perception of mental overload. In addition to these factors, the workers indicate that there are consequences on their health related to exhaustion associated with work performance. However, the variability observed in *Health consequences *responses among these professionals is greater than the two previously commented dimensions.

To set the level of load in each dimension, the scores were compared to the cut-offs established in a prior study that was conducted with a Spanish sample^ (18)^. Contrasting the results obtained with the percentiles of the professional group of Technicians and medium-level professionals, it is observed that *Cognitive demand and complexity of the task* obtains a score close to the 75th percentile, indicating that it is important in mental overload. The remaining dimensions obtained scores under the 75th percentile, but they surpass this level when the typical standard deviation is added. Except for *time management, *these dimensions do not reach the 25th percentile when subtracted. In other words, participants have medium-high mental work load levels. These results partially support the second previously mentioned hypothesis regarding mental overload. It is interesting to emphasise that no differences in this mental load profile in different work conditions were obtained when they were hypothesised to show a priori differences. This result points to the idea of a mental load profile in ICU nurses' work that is independent of other work factors that are distinct from their own functions and tasks. Therefore, to improve and to prevent this situation of mental load, an optimum work position design should be implemented that would avoid simultaneous highly complex tasks and favour an adequate distribution of rest breaks.

A final table shows the relationship between psychosocial factors and mental load. Just as seen in the third hypothesis, the psychosocial factor with a clear cognitive effort component, *psychological demands*, significantly correlates with all of the mental load dimensions. It is also observed that all of the psychosocial factors evaluated correlate with the *Health consequences *dimension, so that the greater the psychosocial risk, the greater the perceived negative consequences on health. This result is in agreement with the Job Demand-Control-Social Support model ^(^ 9 ^)^ and with Imbalance Effort Rewards model ^(^ 10 ^)^, which highlight the influence of psychosocial factors on nurses' health^ (24-25)^.

In addition, it has been observed that the psychosocial factors *Psychological demands*, *Rewards* and *Double shift* explain more than one-third of the subjective mental load. In line with the third hypothesis formulated, the psychosocial factor related to the emotional and cognitive demands of the position is the one that most helps to independently explain overall mental load, which coincides with the empirical and theoretical aspects mentioned ^(^ 1 ^,^ 17 ^)^.

The present work contributes to the development of scientific knowledge, as it contributes novel and prominent information with respect to how ICU nurses value psychosocial factors in their position and also establishes relationships between several psychosocial factors and mental load. Knowing how these professionals experience their activity allows one to design and to set in motion intervention strategies that diminish perceived psychosocial risks, improving the quality of work life.

The strengths of this study should be mentioned: (a) the sample of active professionals makes up almost all of the ICU nurses in the three hospitals with similar characteristics, which allows us to obtain a quite complete representation of the perceived reality; (b) relatively new measuring instruments were utilised that were validated in Chile and have been used in prior investigations in other countries; and (c) the implementation of these instruments was performed through an interview process, which guaranteed a high response rate and reduced the quantity of blank answers. However, limitations were also present. (a) The short version of the SUSESO-ISTAS 21 questionnaire was used. Although this version is valid and reliable and has clear advantages with respect to the time of implementation, it is not recommended for medium- or large-sized organisations, and when factors are grouped, the capacity for obtaining detailed information is limited. (b) As this study was cross-sectional, the results obtained indicate the perception of psychosocial risks and mental work load at a specific time, which impedes the establishment of causal relationships. Nevertheless, the objectives proposed can be undertaken with this type of methodology, as we did not intend to evaluate the development of psychosocial risks and mental work load over time or to value this effect of any type of intervention. (c) One of the dimensions of the SUSESO-ISTAS 21 questionnaire shares some similar items with one of the ESCAM dimensions, which could have influenced the correlations obtained between them. Specifically, the *psychological demands* (SUSESO-ISTAS 21) dimension includes two items out of a total of six items similar to two of those evaluated in the *time management* (ESCAM) dimension, which is composed of three items. No conceptual or operational relationships are observed between the other dimensions of the two instruments.

Future lines of investigation could evaluate prominent psychosocial factors in this study population in more depth. These investigations could include verifying the stability of the mental load profile, studying the effect of one's own emotional load of the position on mental load, studying the influence of mental load on workers objective performance and health data, and developing preventive strategies for these units.

## Conclusions

First, it was determined that inadequate psychosocial factors exist in the ICU, specifically with regard to *psychological demands, double shift *and *social support in the organisation and quality of the leadership.*

Second, it was observed that mental overload for cognitive demands and complexity of tasks exists. The dimensions *Characteristic of the task*, *Work pace* and *Health consequences *produced medium-high mental loads.

Finally, comparison between the dimensions of the instruments utilised show that (a) the *psychological demands* factor positively correlated with all of the dimensions of mental load, and (b) overall mental load is partly explained by the psychosocial factors *psychological demands*, *rewards* and *double* shift.

## Footnotes

[1] Rotating shifts are an internal system of personnel distribution; in this study, it is called the "*fourth shift*", which corresponds to 12 working hours during the day (from 8:00 am to 8:00 pm), followed by a day of 12 working hours at night (from 8:00 pm to 8:00 am), followed by two days free. This rotation is utilised in the three participating units. It should be noted that in Chile, people work 44 hours weekly, independent of the shifts during which the work is performed.
